# Three new species of the genus *Leptonetela* from Greece (Araneae, Leptonetidae)

**DOI:** 10.3897/zookeys.569.6921

**Published:** 2016-02-24

**Authors:** Yi Wu, Chunxia Wang, Guo Zheng, Shuqiang Li

**Affiliations:** 1 College of Life Science, Shenyang Normal University, Shenyang 110034, China; 2 Institute of Zoology, Chinese Academy of Sciences, Beijing 100101, China

**Keywords:** Haplogynae, taxonomy, DNA-barcoding, Balkan Peninsula, spider

## Abstract

Three new species of the spider genus *Leptonetela* collected from caves in Greece are described: *Leptonetela arvanitidisi* **sp. n.** (male & female), *Leptonetela paragamiani* **sp. n.** (male & female) and *Leptonetela penevi* **sp. n.** (male & female). Detailed illustrations of the new species are provided. DNA barcodes were obtained for future use.

## Introduction

The leptonetids are minute (1.0–3.0 mm) spiders that can be easily distinguished from other families by a distinctive 6-eyed pattern, with the posterior median eyes situated behind the posterior lateral eyes; however, in some cave species, the eyes are reduced to vestiges or may be completely absent (Gertsch 1974).

A total of 23 genera and 276 species of the spider family Leptonetidae are known worldwide (World Spider Catalog 2016). The genus *Leptonetela* was established by Kratochvíl (1978), using *Leptonetela kanellisi* (Deeleman-Reinhold, 1971) from a cave in Greece as the type species. *Leptonetela* can be distinguished from other genera of the family by the palpal femur without spines, the retrolateral surface of the palpal tibia with a longitudinal row of strong spines and the male palpal tarsus without appendices.

A total of 50 *Leptonetela* species are known from Europe and Asia. Two species of *Protoleptoneta* were transferred to *Leptonetela* by Brignoli in 1979: *Leptonetela strinatii* (Brignoli, 1976) from Greece and *Leptonetela deltshevi* (Brignoli, 1979) from Turkey. Deltshev described *Leptonetela andreevi* from Greece in 1985. Dunin (1990) reported *Leptonetela caucasica* from Georgia and Azerbaijan. *Leptonetela thracia* was described by Gasparo in 2005 from Greece. Subsquently, Lin and Li (2010) described 24 species occurring in the Yunnan-Guizhou Plateau, China, including *Leptonetela quinquespinata* (Chen & Zhu, 2008) which was transferred from *Qianleptoneta* Chen & Zhu, 2008. Wang and Li (2011) reported 17 *Leptonetela* species from South China, 2 species from Greece and 1 species from Vietnam.

Other than *Leptonetela deltshevi* (Brignoli, 1979) from Turkey and *Leptonetela pungitia* Wang & Li, 2011 from Vietnam which have been collected in epigean habitats, all species are found in caves. Some of them have characters typical to true troglobites, such as lacking eyes and pigmentation and elongated legs.

In this paper, three *Leptonetela* species collected from caves in Greece are described as new to science. The total number of *Leptonetela* species from Europe reaches 9 species.

## Material and methods

Specimens were examined with a LEICA M205C stereomicroscope. Images were captured with an Olympus C7070 wide zoom digital camera (7.1 megapixels) mounted on an Olympus SZX12 dissecting microscope. Epigynes and male palps were examined after dissection from the spiders’ bodies.

Terminology and abbreviations in this paper generally follow Wang and Li (2011) and Ledford (2011). The unit of measurement in this paper is millimetres (mm). Leg metric data were recorded as total length (femur, patella, tibia, metatarsus, tarsus). Leg segments were measured on their dorsal side.

DNA barcodes were obtained for future use. A partial fragment of the mitochondrial gene cytochrome oxidase subunit I (COI) was amplified and sequenced for *Leptonetela arvanitidisi* sp. n., *Leptonetela paragamiani* sp. n. and *Leptonetela penevi* sp. n. following the protocol in Miller et al. (2010). Primers used in this study are: LCO1490 (5’-CWACAAAYCATARRGATATTGG-3’) and HCO-N-2198 (5’- TAAACTTCAGGGTGACCAAAAAATCA -3’) (Folmer et al. 1994). Voucher information and GenBank accession number for all samples are listed in Table 1.

**Table 1.** Voucher specimen information.

Species	Sequence length	Collecting localities	GenBank accession number
*Leptonetela arvanitidisi* sp. n.	620 bp	Greece Athens Attica: Leondari Cave	KU318407
*Leptonetela paragamiani* sp. n.	620 bp	Greece Athens: Pan Cave	KU318410
*Leptonetela penevi* sp. n.	620 bp	Greece Thiva: Skoteini Cave	KU318411

The specimens studied in the current paper are deposited in the Institute of Zoology, Chinese Academy of Sciences (IZCAS) in Beijing, China.

## Taxonomy

### Family Leptonetidae Simon, 1890

#### Genus *Leptonetela* Kratochvíl, 1978

*Leptonetela*: Kratochvíl 1978: 11, f. 1G. Type species *Sulcia kanellisi* Deeleman-Reinhold, 1971 from Greece.

##### Leptonetela arvanitidisi

Taxon classificationAnimaliaAraneaeLeptonetidae

Wang & Li sp. n.

http://zoobank.org/24A56F21-B537-4A53-B19B-53A86AA86C96

Figs 1 , 2 , 7

###### Types.

Holotype ♂ (IZCAS): GREECE, Athens, Attica, Leondari Cave, 37°59'14.61"N, 23°49'47.03"E, elevation 553 m, 28 March 2013, S. Li leg. Paratypes 2♀ (IZCAS), same data as holotype.

###### Etymology.

The specific name is dedicated to Dr. Christos Arvanitidis of the Hellenic Centre for Marine Research in Crete, a leading taxonomist on Polychaeta; noun (name) in genitive case.

###### Diagnosis.

**Figure 1.**
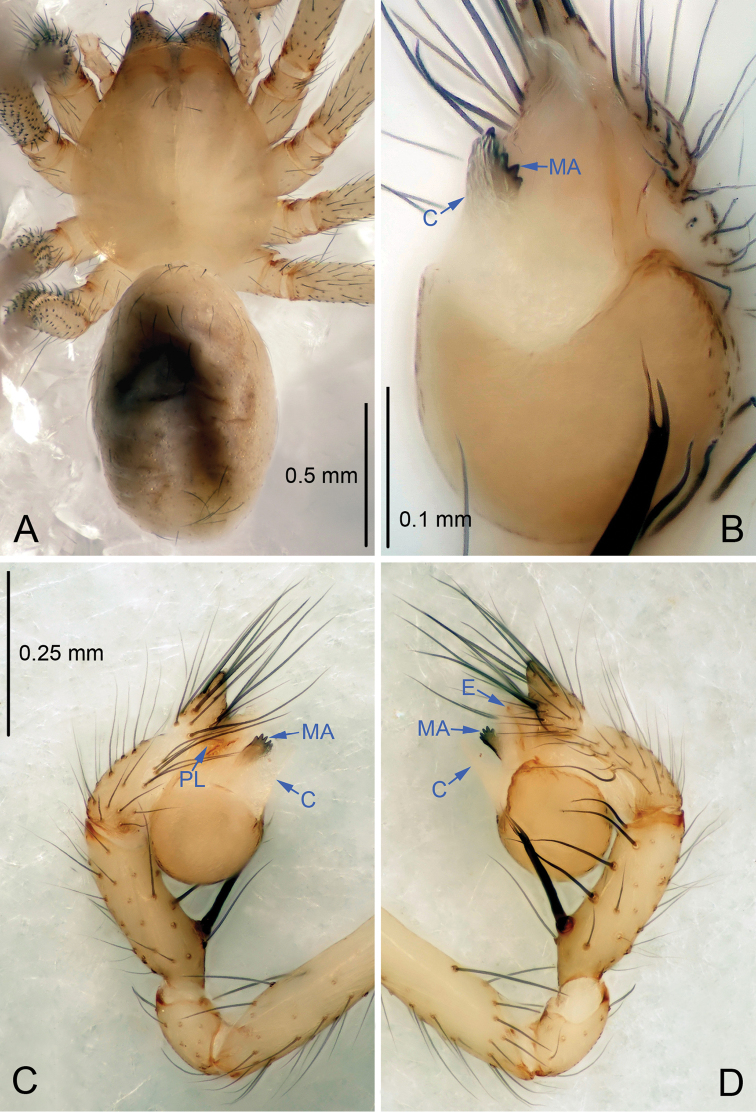
*Leptonetela arvanitidisi* sp. n., holotype male. **A** Habitus, dorsal view **B** Palpal bulb, ventral view **C** Palp, prolateral view **D** Palp, retrolateral view.

**Figure 2.**
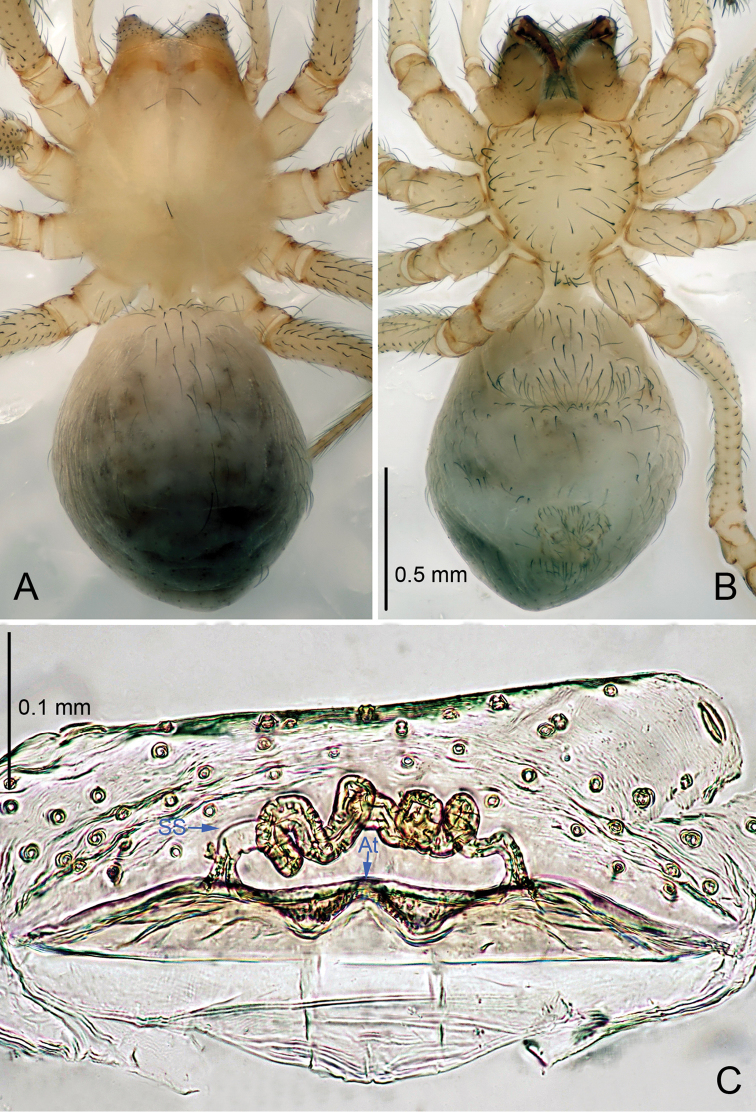
*Leptonetela arvanitidisi* sp. n., one of the paratype female. **A** Habitus, dorsal view **B** Habitus, ventral view **C** Vulva, dorsal view.

*Leptonetela arvanitidisi* sp. n. is similar to *Leptonetela kanellisi* but can be separated by the basal tibial spine with bifurcated tip (Fig. 1D) (not bifurcated in *Leptonetela kanellisi*), the wave-shaped anterior margin of the atrium and the tightly twisted spermathecae (Fig. 2C); *Leptonetela kanellisi* has an arc-shaped anterior margin of the atrium and the spermathecae are loosely twisted (see Wang and Li 2011: figs 16–19).

###### Description.

Male (holotype). Total length 1.75 (Fig. 1A). Carapace 0.83 long, 0.72 wide. Opisthosoma 1.00 long, 0.65 wide. Prosoma yellowish, with one seta in the middle of the carapace. Ocular area with a pair of setae, eyes absent. Median groove, cervical grooves and radial furrows indistinct. Clypeus 0.10 high. Sternum and legs yellowish. Opisthosoma pale brown, ovoid, lacking distinctive pattern. Leg measurements: I 8.39 (2.25, 0.38, 2.45, 2.08, 1.23); II 7.14 (1.88, 0.38, 2.03, 1.72, 1.13); III 5.93 (1.73, 0.35, 1.62, 1.55, 0.68); IV 7.83 (2.13, 0.38, 2.15, 2.00, 1.17). Male palp (Fig. 1C–D): tibia with 5 spines retrolaterally, the basal one strong, conspicuous, with bifurcated tip. Bulb with triangular embolus; prolateral lobe oval. Median apophysis (Fig. 1B) distal edge round, with six small teeth. Conductor membranous, triangular in ventral view.

Female (one of the paratypes). Similar to male in color and general features but larger and with shorter legs. Total length 2.03 (Fig. 2A–B). Carapace 0.85 long, 0.73 wide. Opisthosoma 1.23 long, 0.90 wide. Clypeus 0.10 high. Leg measurements: I 7.24 (1.90, 0.38, 2.08, 1.75, 1.13); II 5.92 (1.68, 0.33, 1.63, 1.38, 0.90); III 5.32 (1.50, 0.32, 1.42, 1.30, 0.78); IV 6.63 (1.75, 0.35, 1.80, 1.70, 1.03). Vulva (Fig. 2C): spermathecae coiled, atrium fusiform, anterior margin of the atrium wave shaped.

###### Distribution.

Known only from the type locality.

##### Leptonetela paragamiani

Taxon classificationAnimaliaAraneaeLeptonetidae

Wang & Li sp. n.

http://zoobank.org/28C5914F-58AB-409B-8110-61F5B1D5004D

Figs 3 , 4 , 7

###### Types.

Holotype ♂ (IZCAS): GREECE, near Athens, Pan Cave, 38°08'48.54"N, 23°40'06.04"E, elevation 660 m, 7 April, 2013, S. Li leg. Paratypes 2 ♀ (IZCAS), same data as holotype.

###### Etymology.

The specific name is dedicated to Mr. Kaloust Paragamian of the Hellenic Institute of Speleological Research in Crete, a leading speleologist in Greece; noun (name) in genitive case.

###### Diagnosis.

**Figure 3.**
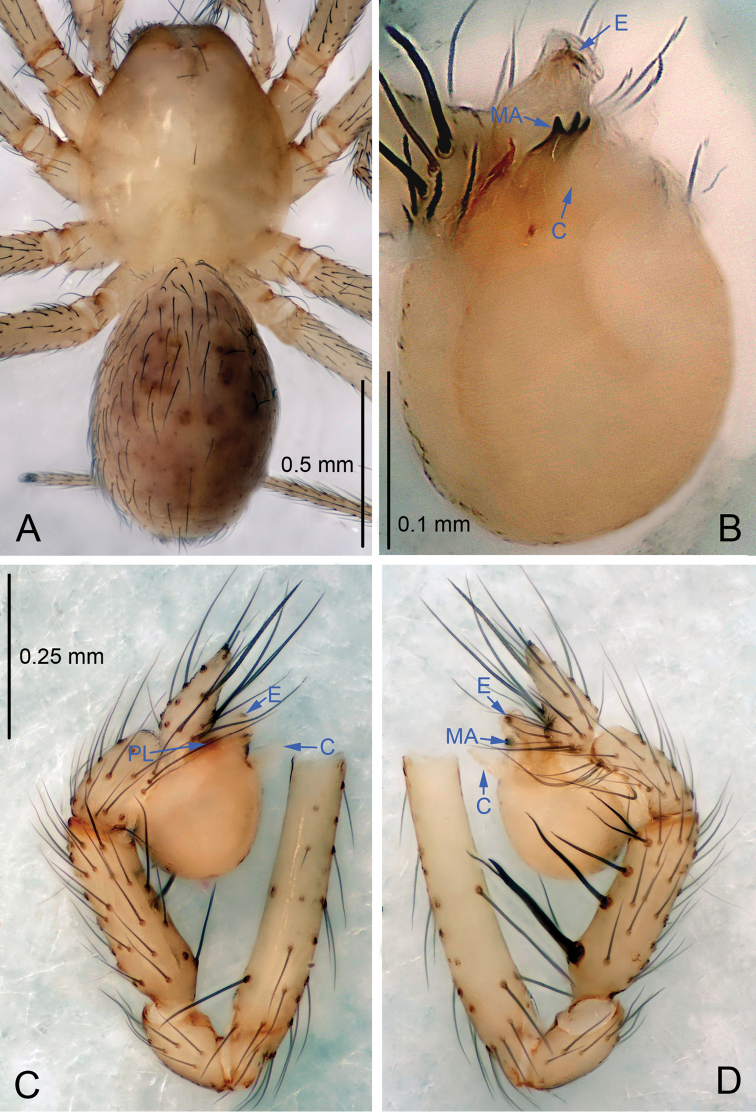
*Leptonetela paragamiani* sp. n., holotype male. **A** Habitus, dorsal view **B** Palpal bulb, ventral view **C** Palp, prolateral view **D** Palp, retrolateral view.

**Figure 4.**
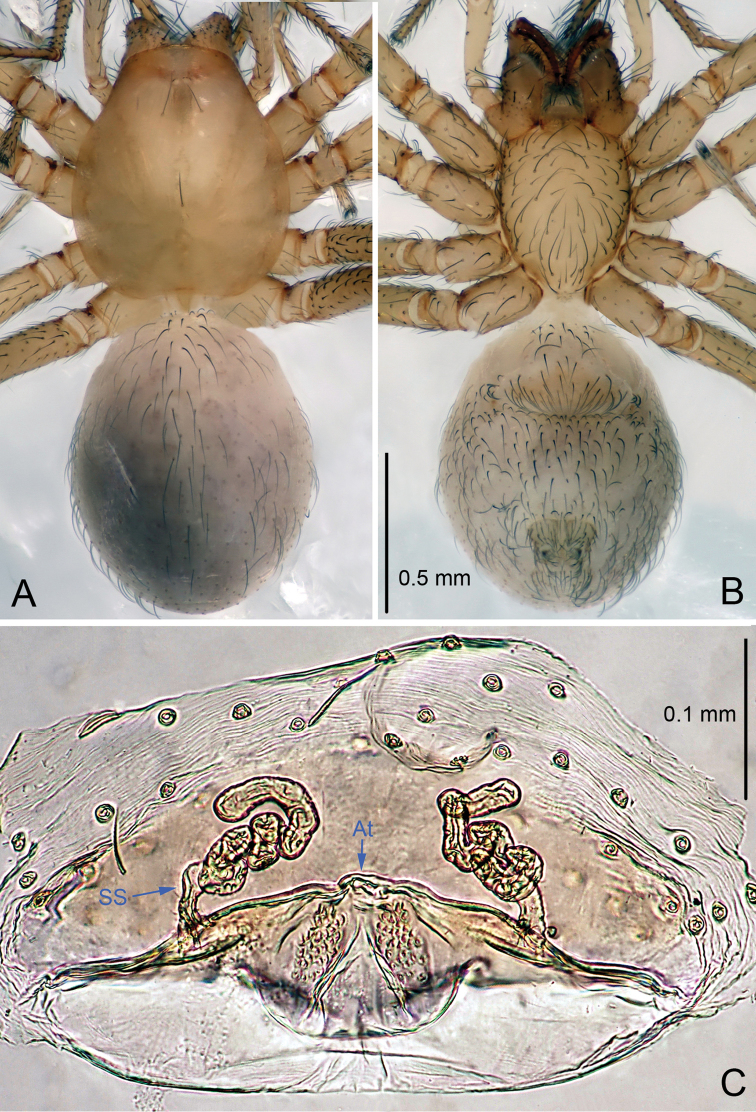
*Leptonetela paragamiani* sp. n., one of the paratype female. **A** Habitus, dorsal view **B** Habitus, ventral view **C** Vulva, dorsal view.

*Leptonetela paragamiani* is similar to *Leptonetela kanellisi* and *Leptonetela arvanitidisi* sp. n. but can be separated by the second tibial spine, which is longest in *Leptonetela paragamiani* sp. n., whereas in *Leptonetela kanellisi* and *Leptonetela arvanitidisi* sp. n. (Fig. 3D) the basal spine is longest; the median apophysis has 3 small teeth (Fig. 3B) in *Leptonetela paragamiani* sp. n., whereas it has 6 teeth in *Leptonetela kanellisi* and *Leptonetela arvanitidisi* sp. n.; and the spermathecae are tightly twisted (Fig. 4C) compared to the spermathecae of *Leptonetela kanellisi* and *Leptonetela arvanitidisi* sp. n.

###### Description.

Male (holotype). Total length 1.63 (Fig. 3A). Carapace 0.75 long, 0.62 wide. Opisthosoma 0.88 long, 0.62 wide. Prosoma yellowish, with one seta on the median part. Ocular area with a pair of setae, two eyes, reduced to white spots. Median groove, cervical groove and radial furrows indistinct. Clypeus 0.10 high. Sternum and legs yellowish. Opisthosoma pale brown, ovoid, lacking distinctive pattern. Leg measurements: I 5.53 (1.50, 0.28, 1.60, 1.27, 0.88); II 4.78 (1.38, 0.25, 1.27, 1.13, 0.75); III 4.01 (1.13, 0.25, 1.03, 1.00, 0.60); IV 5.25 (1.45, 0.28, 1.47, 1.25, 0.80). Male palp (Fig. 3C–D): tibia with 5 retrolateral spines, the basal one strong, conspicuous, and the second one longer than others. Bulb with spoon-shaped embolus, prolateral lobe oval. Distal edge of median apophysis round (Fig. 3B), with three small teeth, conductor membranous, shield shaped in ventral view.

Female (one of the paratypes). Similar to male in color and general features but larger and with longer legs. Total length 1.88 (Fig. 4A–B). Carapace 0.75 long, 0.68 wide. Opisthosoma 1.00 long, 0.88 wide. Clypeus 0.10 high. Leg measurements: I 6.26 (1.75, 0.28, 1.80, 1.53, 0.90); II 5.36 (1.58, 0.28, 1.50, 1.25, 0.75); III 4.69 (1.38, 0.25, 1.25, 1.13, 0.68); IV 6.19 (1.78, 0.28, 1.75, 1.50, 0.88). Vulva (Fig. 4C): spermathecae twisted, atrium oval.

###### Distribution.

Known only from the type locality.

##### Leptonetela penevi

Taxon classificationAnimaliaAraneaeLeptonetidae

Wang & Li sp. n.

http://zoobank.org/C819DE91-5E16-4B18-B336-26AE51FFD15F

Figs 5 , 6 , 7

###### Types.

Holotype ♂ (IZCAS): GREECE, Thiva, Kakalitsa, Skoteini Cave, 38°29'59.81"N, 23°59'01.06"E, elevation 443 m, 29 March, 2013, S. Li leg. Paratypes 2 ♀, same data as holotype.

###### Etymology.

The specific name is dedicated to Prof. Dr. Lyubomir Penev, zoologist and founder of Pensoft Publishers; noun (name) in genitive case. Pensoft Publishers is a leading company in publishing taxonomic works.

###### Diagnosis.

**Figure 5.**
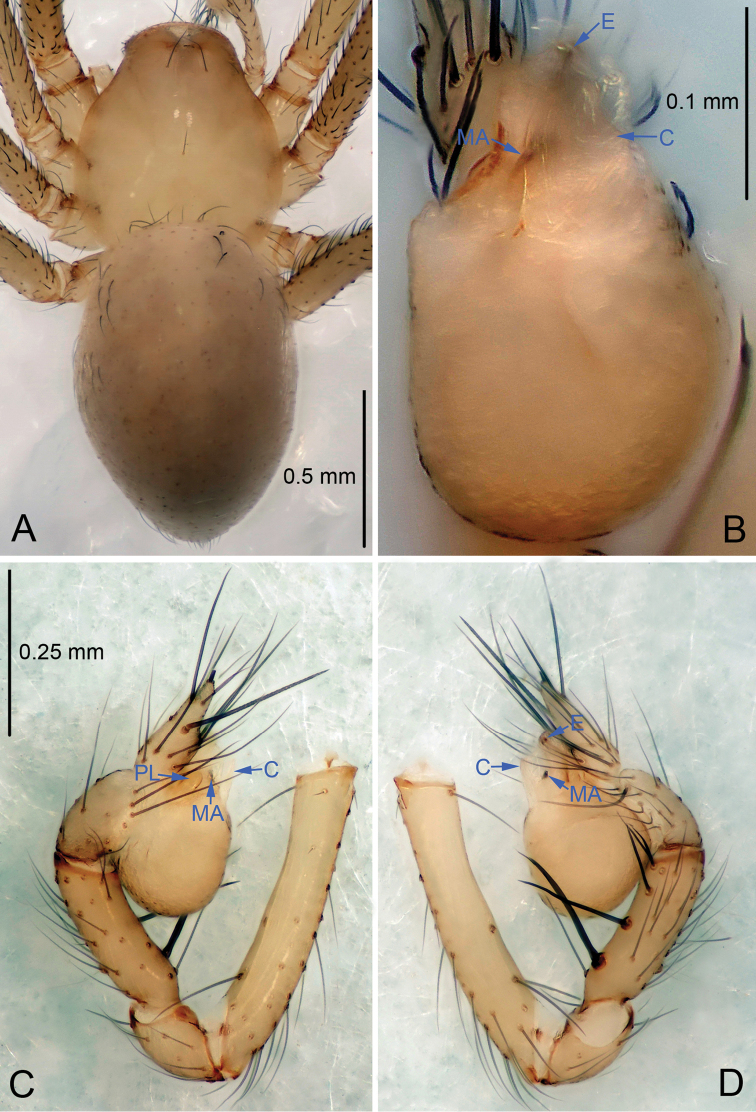
*Leptonetela penevi* sp. n., holotype male. **A** Habitus, dorsal view **B** Palpal bulb, ventral view **C** Palp, prolateral view **D** Palp, retrolateral view.

**Figure 6.**
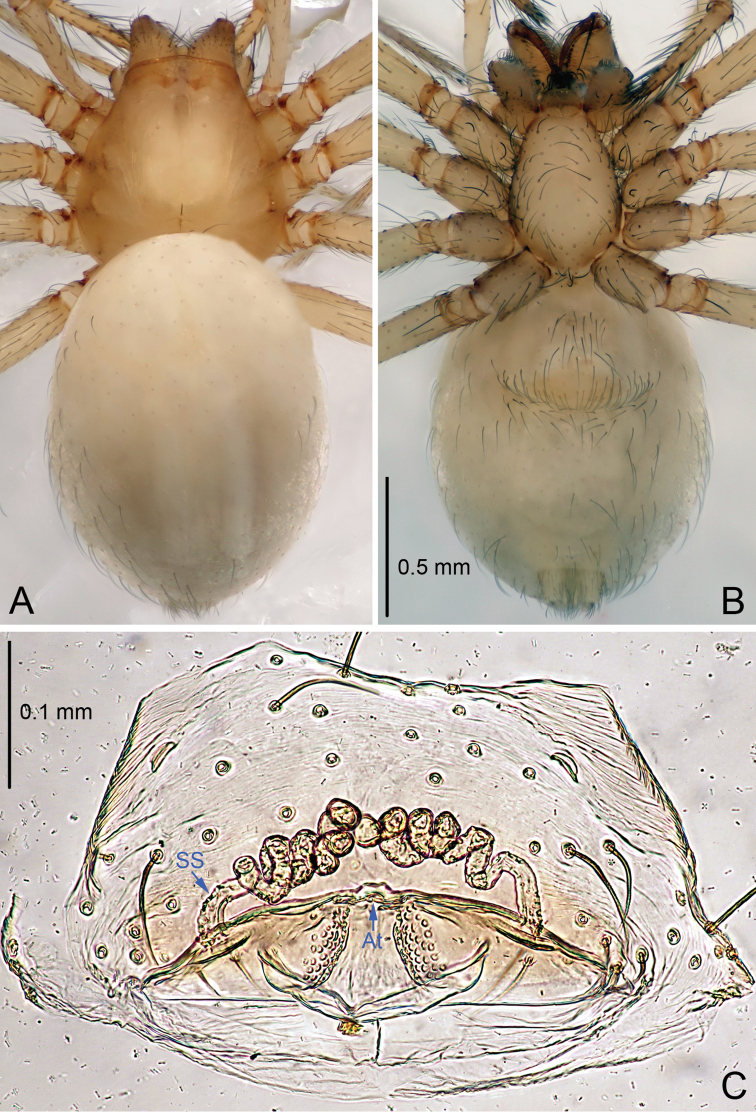
*Leptonetela penevi* sp. n., one of the paratype female. **A** Habitus, dorsal view **B** Habitus, ventral view **C** Vulva, dorsal view.

*Leptonetela penevi* sp. n. is similar to *Leptonetela kanellisi* and *Leptonetela paragamiani* sp. n. but can be separated by having the basal tibial spine longer than others, and slender (Fig. 5D) compared to the basal spines of *Leptonetela kanellisi* and *Leptonetela paragamiani* sp. n.; median apophysis distally without teeth (Fig. 5D) and spermathecae strongly twisted and longer than those of *Leptonetela kanellisi* and *Leptonetela paragamiani* sp. n. (Fig. 6C).

###### Description.

Male (holotype). Total length 1.83 (Fig. 6A). Carapace 0.77 long, 0.65 wide. Opisthosoma 1.15 long, 0.80 wide. Prosoma yellowish, with one seta on the median part. Ocular area with a pair of setae, eyes absent. Median groove, cervical groove and radial furrows indistinct. Clypeus 0.10 high. Sternum and legs yellowish. Opisthosoma pale brown, ovoid, lacking distinctive pattern. Leg measurements: I 6.76 (1.88, 0.38, 1.87, 1.55, 1.08); II 5.44 (1.38, 0.33, 1.58, 1.27, 0.88); III 4.87 (1.37, 0.30, 1.25, 1.20, 0.75); IV 6.32 (1.82, 0.35, 1.73, 1.50, 0.92). Male palp (Fig. 5C–D): tibia with 5 spines retrolaterally, with the basal one strong, conspicuous, and longest. Bulb oval, with spoon-shaped embolus, prolateral lobe oval. Median apophysis (Fig. 5B) without teeth distally, conductor membranous, rugose and shield shaped in ventral view.

Female (one of the paratypes). Similar to male in color and general features but larger and with shorter legs. Total length 2.03 (Fig. 6A–B). Carapace 0.75 long, 0.72 wide. Opisthosoma 1.38 long, 0.85 wide. Clypeus 0.10 high. Leg measurements: I 6.51 (1.88, 0.38, 1.83, 1.50, 0.92); II 5.54 (1.63, 0.33, 1.55, 1.25, 0.78); III 4.91 (1.42, 0.33, 1.28, 1.13, 0.75); IV 6.31 (1.80, 0.35, 1.80, 1.48, 0.88). Vulva (Fig. 6C): spermathecae strongly twisted, atrium oval.

###### Distribution.

Known only from the type locality.

**Figure 7.**
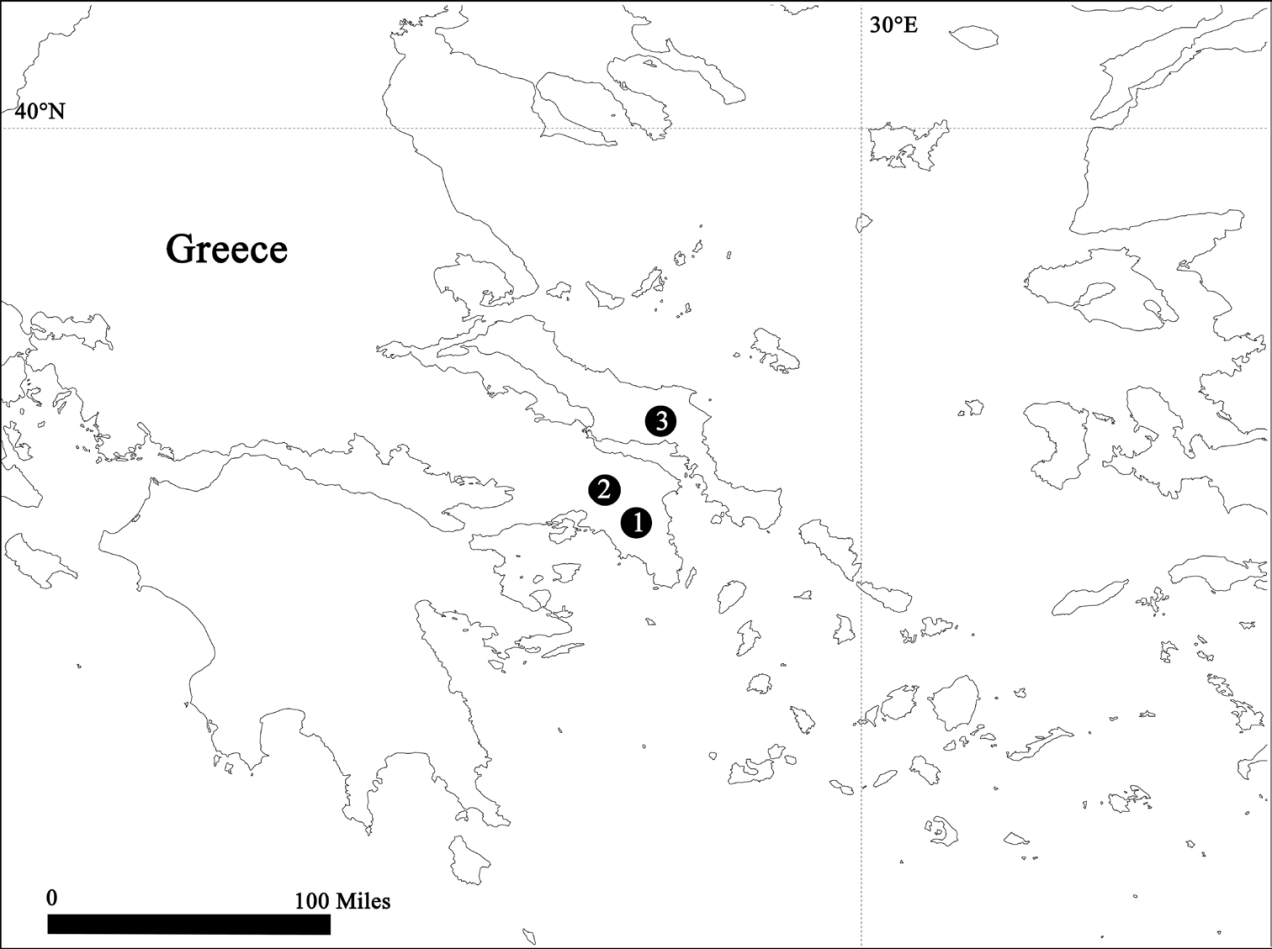
Locality records for three new species of *Leptonetela* in Greece: ① *Leptonetela arvanitidisi* sp. n. (Athens) ② *Leptonetela paragamiani* sp. n. (near Athens) ③ *Leptonetela penevi* sp. n. (Thiva).

## Supplementary Material

XML Treatment for Leptonetela arvanitidisi

XML Treatment for Leptonetela paragamiani

XML Treatment for Leptonetela penevi

## References

[B1] Brignoli PM (1979) Spiders from Turkey, VI. Four new species from the coast of the Black Sea (Araneae). Bulletin of the British Arachnological Society 4: 310–313.

[B2] Deltshev CD (1985) New data concerning cave spiders (Araneae) in Greece with description of a new *Leptonetela* (Araneae, Leptonetidae). Acta Zoologica Bulgarica 27: 41–45.

[B3] Dunin PM (1990) *Leptonetela caucasica* sp. n. - a first finding of spiders of the family Leptonetidae (Aranei, Haplogynae) in the USSR. Zoologicheskiĭ Zhurnal 69(1): 147–149.

[B4] Folmer O Black M Hoeh W Lutz R Vrijenhoek R (1994) DNA primers for amplification of mitochondrial cytochrome coxidase subunit I from diverse metazoan invertebrates. Molecular Marine Biology and Biotechnology 3(5): 294–299. 7881515

[B5] Gasparo F (2005) Una nuova *Leptonetela cavernicola* di Grecia (Araneae, Leptonetidae). Bollettino del Museo Regionale di Scienze Naturali di Torino 22: 517–524.

[B6] Gertsch WJ (1974) The spider family Leptonetidae in North America. The Journal of Arachnology, 45–203.

[B7] Kratochvíl J (1978) Araignées cavernicoles des îles Dalmates. Přírodovědné práce ústavů Československé akademie věd v Brně (N. S.) 12(4): 1–59.

[B8] Ledford J Paquin P Cokendolpher J Campbell J Griswold C (2011) Systematics of the spider genus *Neoleptoneta* Brignoli, 1972 (Araneae: Leptonetidae) with a discussion of the morphology and relationships for the North American Leptonetidae. Invertebrate Systematics 25: 334–388. doi: 10.1071/IS11014

[B9] Lin Y Li S (2010) Leptonetid spiders from caves of the Yunnan-Guizhou plateau, China (Araneae: Leptonetidae). Zootaxa 2587: 1–93.

[B10] Miller JA Carmichael A Ramirez MJ Spagna JC Haddad CR Řezáč M Johannesen J Král J Wang XP Griswold CE (2010) Phylogeny of entelegyne spiders: Affinities of the family Penestomidae (NEW RANK), generic phylogeny of Eresidae, and asymmetric rates of change in spinning organ evolution (Araneae, Araneoidea, Entelegynae). Molecular Phylogenetics and Evolution 55: 786–804. doi: 10.1016/j.ympev.2010.02.021 20206276 10.1016/j.ympev.2010.02.021

[B11] Wang C Li S (2011) A further study on the species of the spider genus *Leptonetela* (Araneae: Leptonetidae). Zootaxa 2841: 1–90.

[B12] World Spider Catalog (2016) World Spider Catalog. Natural History Museum Bern Version 17.0. http://wsc.nmbe.ch [accessed 29 January 2016]

